# Thyroid disturbances after COVID-19 and the effect of vaccination in children: a prospective tri-center registry analysis

**DOI:** 10.1007/s00431-023-05097-8

**Published:** 2023-07-25

**Authors:** Vivien Herczeg, Réka Garai, Johanna Takács, Fanni Kovács, Andrea Luczay, Erzsébet Hrapka, Péter Krivácsy, Éva Hosszú, Nikolett Jusztina Beniczky, Ágnes Németh, Eszter Szabina Szilágyi, Anna Pécsi, Zsófia Szabó, Attila József Szabó, Péter Tóth-Heyn

**Affiliations:** 1 1st Department of Paediatrics, Semmelweis University, Budapest, Hungary; 2 Pediatric Center, MTA Center of Excellence, Semmelweis University, Bókay Unit, Bókay János Street 53-54, 1083 Budapest, Hungary; 3 Centre for Translational Medicine, Semmelweis University, Budapest, Hungary; 4 Department of Social Sciences, Faculty of Health Sciences, Semmelweis University, Budapest, Hungary; 5 2nd Department of Paediatrics, Semmelweis University, Budapest, Hungary; 6 Faculty of Medicine, Semmelweis University, Budapest, Hungary; 7 Department of Laboratory Medicine, Semmelweis University, Budapest, Hungary; 8 ELKH-SE Pediatrics and Nephrology Research Group, Budapest, Hungary

**Keywords:** COVID-19 vaccines, Hashimoto disease, Pediatrics, Post-acute COVID-19 syndrome, SARS-CoV-2, Thyroiditis, autoimmune

## Abstract

Rapidly evolving clinical data suggest that the novel coronavirus (SARS-CoV-2) and vaccination against COVID-19 might be associated with thyroid disturbances. However, studies remain limited among the pediatric population**.** Our aim was to assess the prevalence and permanence of thyroid autoimmunity (TA) and dysfunction in children after an acute infection and its potential association with vaccination. A prospective, multicenter registry analysis was performed among 458 children (mean age: 12.4 ± 3,8 years, 45.4% male) with preceding COVID-19. Patient inclusion lasted from 24^th^ March, 2021 to 23^rd^ March, 2022 at three pediatric outpatient facilities at Semmelweis University, Budapest. Primary outcomes were the rate of thyroid disturbances assessed by laboratory parameters (thyroid function tests, antithyroglobulin [ATG] and anti-thyroid peroxidase [ATPO] antibodies) and thyroid ultrasound. TA rate among vaccinated and unvaccinated children was determined. Children with newly diagnosed thyroid alterations were followed up for 12.7 ± 4.3 months. Six children had previous thyroid disease. Out of 452 children, 30 cases (6.6%) of newly diagnosed TA (six of them had abnormal thyroid-stimulating hormone [TSH] levels) and eight cases (1.8%) of isolated TSH elevation were observed. Ultrasound-proven autoimmune thyroiditis (AIT) was 4.0%. No association was found between COVID-19 vaccination and thyroid autoimmunity (χ^2^(1,N = 452) = 0.138, *p* = 0.815). Among children with TA, 73.3% had long-lasting alterations.

*  Conclusion*: Vaccination had no effect on the prevalence of TA. Until further controlled studies state otherwise, children with preceding COVID-19 might benefit from thyroid screening.

**Table:** 

**What is Known:** *• Numerous case reports implicate that coronavirus disease-2019 (COVID-19) and vaccination against SARS-CoV-2 can be responsible for thyroid disturbances.* *• Thyroid alterations discovered during acute COVID-19 tend to cease by time and only incidental thyroid autoimmunity (TA) is diagnosed after COVID-19. In adults, no increase in vaccine-related hyper- or hypothyroidism was found.*	
**What is New:** *• TA rate after COVID-19 vaccination among children was not increased. TA had no role in long COVID syndrome.* *• We discovered a considerable rate of TA (6.6%) and ultrasound-proven autoimmune thyroiditis (AIT) (4.0%) after SARS-CoV-2 infection, and the majority of these alterations remained positive after 6 months.*

**What is Known:**

*• Numerous case reports implicate that coronavirus disease-2019 (COVID-19) and vaccination against SARS-CoV-2 can be responsible for thyroid disturbances.*

*• Thyroid alterations discovered during acute COVID-19 tend to cease by time and only incidental thyroid autoimmunity (TA) is diagnosed after COVID-19. In adults, no increase in vaccine-related hyper- or hypothyroidism was found.*

**What is New:**

*• TA rate after COVID-19 vaccination among children was not increased. TA had no role in long COVID syndrome.*

*• We discovered a considerable rate of TA (6.6%) and ultrasound-proven autoimmune thyroiditis (AIT) (4.0%) after SARS-CoV-2 infection, and the majority of these alterations remained positive after 6 months.*

## Introduction

A growing body of research has raised awareness of a possible bidirectional connection between coronavirus disease 2019 (COVID-19) and thyroid dysfunction. The novel coronavirus (SARS-CoV-2) might trigger or accelerate autoimmunity as reactivation of previous autoimmune thyroid disorders along with a new onset Hashimoto and Graves’ disease after COVID-19 were observed [1, 2]. Appearance and increasing titers of anti-thyroid peroxidase (ATPO) and radiological signs of thyroid autoimmunity (TA) were also reported by several studies during the acute phase and at follow-up of COVID-19 patients [3–5]. However, Lui et al. found only incidental ATPO and antithyroglobulin (ATG) positivity among 246 SARS-CoV-2 survivors at three and 6 months of follow-up [6]. Additionally, a remarkable amount of reports described subacute thyroiditis (SAT) and autoimmune thyroid disturbances after the application of various COVID-19 vaccines [7–9]. Children are generally more protected from the complications of the acute disease; however, long-term consequences (e.g., long COVID syndrome [LCS]) may have a significant impact on their lives; thus, there is a huge need to expand the borders of our knowledge [10]. Considering the thyroid gland, data on the pediatric population is scarce and controversial.

The importance of the recognition and management of overt hormonal complications, as well as early identification of thyroiditis to get ahead of developing malignancies is indisputable. Based on our initial clinical experience of detecting high frequency of positive thyroid autoantibodies in children presenting to the Long COVID outpatient facility of the 1^st^ Department of Paediatrics, Semmelweis University [10]; in our prospective study, we aim to evaluate (i) the rate of abnormal thyroid function tests, anti-thyroid antibody positivity and ultrasound-proven thyroiditis in post-COVID period, (ii) the association between COVID-19 vaccination (potential pathogenic or preventive role) and thyroid autoimmunity, (iii) the association between LCS and thyroid autoimmunity and (iv) the longitudinal changes of the initial anti-thyroid positivity and abnormal thyroid function.

## Materials and methods

### Study design and setting

Data were collected in a prospective registry (Hungarian Pediatric Long COVID Registry) with standardized data from three pediatric Long COVID (LC) outpatient clinics of Semmelweis University, Budapest, Hungary. Children showing no LC symptoms were invited to participate in our endocrine screening at the outpatient facility of the 1^st^ Department of Paediatrics for research purposes, were also involved in the study. The first visits were carried out between 24^th^ March, 2021 (the opening of the first pediatric LC clinic at Semmelweis University) and 23^rd^ March, 2022. Answers given to a detailed questionnaire by the parents were verified by a medical doctor. Physical examination and laboratory testing were also accomplished during this in-person visit. We followed a standardized laboratory testing protocol based on the available World Health Organization (WHO) guideline (https://www.who.int/publications/i/item/global-covid-19-clinical-platform-case-report-form-(crf)-for-post-covid-conditions-(post-covid-19-crf-), accessed: 03.04.2023); therefore, a measurement of thyroid parameters was executed in all children regardless of their symptoms. In case of any abnormal thyroid values, the patient was referred to a pediatric endocrinologist. In all cases when the specialists did not indicate further tests, we contacted the families through a phone call and invited them to participate in the follow-up. Reassessments were executed after a minimum of two and a maximum of 19 months and were included in the present analysis until 23^rd^ of December, 2022.

### Inclusion and exclusion criteria, patient groups

**Fig. 1:**
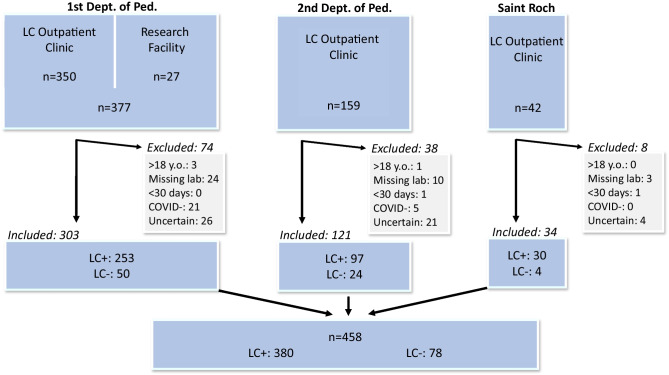
Flowchart of patient inclusion. LC: Long COVID. LC + : children who had one or more new and/or worsened symptoms since their COVID-19 which was still present at the time of their examination (long COVID syndrome). LC-: children who had confirmed COVID-19 but were not experiencing long-lasting symptoms in connection with the previous infection and were complaint-free at the time of their first visit

We included all children with prior SARS-CoV-2 infection proven by positive polymerase chain reaction (PCR) and/or rapid antigen (RAT) and/or serology (anti-spike and/or anti-nucleocapsid antibodies) tests (See Fig. 1.). Anti-spike antibody positivity was only recognised as a proof of previous COVID-19 in non-vaccinated children. However, since the presence of viral nucleocapsid antibody is not affected by vaccinations, its positivity was considered as an indication of a preceding infection. Exclusion criteria can be seen in Fig. 1. Enrolled children were further divided into two groups: children with persisting symptoms after COVID-19 (LCS, LC + group) and children who previously had confirmed COVID-19 but were complaint-free at our examination (LC- group).

 > 18. y.o.: Subjects ≥ 18 years. Missing lab: Children who had one or more missing thyroid laboratory values. < 30 days: Children whose examinations were performed less than 30 days after their coronavirus infection. COVID-: Children who did not have a confirmed acute infection and their serology tests were negative. Uncertain: Children whose infection could not be proven with certainty because they had already been vaccinated before their anti-spike serology test and did not have an anti-nucleocapsid antibody result.

### Measured parameters

Collected parameters were: date of visits, clinical data of children (sex, date of birth, height, weight, history of previous thyroid disease), acute SARS-CoV-2 infection (date, method of proof, severity), COVID-19 vaccination status and date, persisting symptoms, thyroid laboratory values (ATPO, ATG, TSH, and fT4 if the TSH was altered). BMI Z-scores were determined based on the national longitudinal age-specific child-growth report [11]. Severity was assessed according to the WHO’s classification (https://www.who.int/publications/i/item/WHO-2019-nCoV-clinical-2021-2, accessed: 03.04.2023). Laboratory assay methodology and ranges of normal values can be seen in Table 1. Thyroid autoimmunity (TA) was defined as the presence of at least one positive thyroid autoantibody. Autoimmune thyroiditis (AIT) was determined by at least one positive autoantibody and ultrasound-proven thyroiditis. Thyroid ultrasounds were considered as positive for thyroiditis in case of enlargement and/or inhomogeneity and/or hyperaemia of the thyroid gland. The necessity and type of medication (hormone replacement or antithyroid therapy) was recorded as well. Long-lasting alteration was defined by any of the non-physiologic parameters (ATPO and/or ATG and/or ultrasound positivity and/or TSH abnormality) remaining altered at least 6 months after the initial assessment. When parameters normalized within 6 months, we considered the changes as transient.

**Table 1:** Laboratory assay methodology and ranges of normal values

	Assay methodology	Units of measurement	Lower limit	Upper limit
Anti-thyroid peroxidase (ATPO) *until 11th of January, 2022*	CLIA, Abbott	U/mL	-	5.6
Anti-thyroid peroxidase (ATPO) *from 12th of January, 2022*	ECLIA, Roche	U/mL	9	34
Antithyroglobulin (ATG)	ECLIA, Roche	UI/mL	-	115
TSH receptor antibodies (TRAb)	ECLIA, Roche	IU/L	-	1.75
Thyroid-stimulating hormone (TSH)	CLIA, Siemens	mU/L	0.35	4.94
Free thyroxine (fT4)	CLIA, Siemens	pmol/L	9	23.2
Anti-SARS-CoV-2 spike antibody	ECLIA (Elecsys® Anti-SARS-CoV-2 S assay, Roche), reference number: 09289267190	U/mL	-	0.8
Anti-SARS-CoV-2 nucleocapsid antibody	ELISA (GA CoV-2 IgG + , Generic Assay), reference number: 3940	BI (binding index)	-	1.2

*CLIA* Chemiluminescence Immunoassay, *ECLIA* Electro-chemiluminescence Immunoassay, *ELISA* Enzyme-linked Immunosorbent Assay

### Data extraction and statistical analysis

Clinical data were stored in REDCap electronic data capture tools hosted at Semmelweis University [12, 13] and laboratory results were obtained from the institutional Medsol software. Descriptive statistics were reported in mean, standard deviation and relative frequencies. To examine group differences, independent samples *t*-test was used, and for testing association, Fisher’s exact test was applied with phi coefficient as the measure of association. As the autoantibody levels followed log-normal distributions, we used the base ten logarithm of the values for plots and for estimating the mean effect. For the estimation of the mean effect of time on autoantibody value changes, a generalized least square method was used with linear relation and autoregressive correlation structure assumptions. The model fit was assumed with simulation diagnostic plots using DHARMa package. Statistical calculations were made by IBM SPSS Statistics for Windows, Version 28.0 (IBM Corp. Released 2021. Armonk, NY: IBM Corp), and R software (v4.2.1 [14]) using packages nlme (v3.1.157 [15]), DHARMa (R-DHARMa) for calculations and ggplot2 (v3.3.6 [16]) for plotting the results.

## Results

### Demographics

From 24^th^ March, 2021 to 23^rd^ March, 2022, we examined 578 children in the three outpatient facilities. After exclusions, a total of 458 children were enrolled in our analysis (See Fig. 1). Mean age was 12.4 ± 3.8 yrs, and 208 (45.4%) were male. All but one patient belonged to the Caucasian ethnicity. Mean height was 154.2 ± 22.1 cm, mean weight 47.2 ± 18.6 kg, and mean BMI Z score 0.1 ± 1.2.

### SARS-CoV-2 testing and severity of the acute infection

In 290 cases, the date of COVID-19 was proven by PCR and/or RAT. In the other cases, antibody tests confirmed a previous infection. The severity of acute COVID-19 based on WHO criteria was asymptomatic or mild in most of the cases (91.3%, *n* = 418), while in 35 (7.6%) children, the severity of acute infection was moderate, and it was unknown in 5 cases.

### Previous thyroid disease

Six children had a known history of thyroid disease or thyroid laboratory alterations before their SARS-CoV-2 infection. Five of them had laboratory alterations at their first visit. Their details can be seen in Table 2.

**Table 2:** Laboratory values, ultrasound results, and need for medication at the first evaluation of children with thyroid disturbances

	Sex	Age (years)	Absolute ATPO (U/mL)	Relative ATPO	Absolute ATG (UI/mL)	Relative ATG	TSH (mU/L)	fT4 (pmol/L)	Thyroid ultrasound	Medication
*Children with newly diagnosed thyroid autoimmunity*										
1	M	10.8	**229.72**	**41.02**	**194.40**	**1.69**	**5.83**	12.09	**Positive**	-
2	F	10.7	**38.47**	**6.87**	**4004.00**	**34.82**	**9.54**	10.86	**Positive**	Levothyroxine
3	F	8.4	0.16	0.03	**508.60**	**4.42**	3.79		**Positive**	-
4	F	15.5	**29.70**	**5.30**	61.73	0.54	1.06		Negative	-
5	F	6.4	0.16	0.03	**468.20**	**4.07**	1.98		Negative **(*Positive*)**	-
6	F	8.4	**77.27**	**13.80**	**258.30**	**2.25**	1.85		**Positive**	-
7	F	14.8	**207.58**	**37.07**	**1121.00**	**9.75**	2.11		**Positive**	-
8	M	12.0	**11.70**	**2.09**	74.05	0.64	2.21		**Positive**	-
9	M	14.5	**135.50**	**24.20**	**371.70**	**3.23**	4.60		**Positive**	-
10	F	13.6	**125.53**	**22.42**	34.41	0.30	1.94		**Positive**	-
11	M	17.6	**5.71**	**1.02**	17.38	0.15	**0.25**	14.48	Negative	-
12*	F	16.1	**3111.10**	**555.55**	**810.50**	**7.05**	** < 0.002**	**33.90**	**Positive**	Thiamazole
13	F	15.5	**254.00**	**7.47**	**4000.00**	**34.78**	1.01		**Positive**	-
14	F	13.1	**232.00**	**6.82**	**126.00**	**1.10**	1.63		Negative	-
15	F	11.6	9.03	0.27	**132.00**	**1.15**	0.94		Negative	-
16	M	15.8	**6.00**	**1.07**	13.14	0.11	**0.16**	11.74	Negative	-
17	F	12.9	**8.75**	**1.56**	**187.60**	**1.63**	1.74		**Positive**	-
18	F	5.9	**7.63**	**1.36**	17.59	0.15	2.03		Negative	-
19	F	16.0	0.83	0.15	**229.00**	**1.99**	0.80		Negative	-
20	F	14.3	4.29	0.77	**338.70**	**2.95**	1.36		Negative	-
21	F	14.3	**305.56**	**54.56**	**427.50**	**3.72**	2.81		**Positive**	-
22	F	15.5	**46.80**	**1.38**	**329.00**	**2.86**	0.91		**Positive**	-
23	F	8.3	**817.76**	**146.03**	**344.80**	**3.00**	3.01		**Positive**	- (*Levothyroxine*)
24	F	13.8	**273.83**	**48.90**	**620.20**	**5.39**	**7.86**	13.16	**Positive**	Levothyroxine
25	F	14.3	**193.45**	**34.54**	**586.50**	**5.10**	1.64		Negative **(*Positive*)**	-
26	F	13.6	**167.00**	**4.91**	41.20	0.36	1.87		Negative	-
27	F	15.1	**10.13**	**1.81**	31.27	0.27	1.32		Negative	-
28	M	16.8	**6.35**	**1.13**	16.83	0.15	2.48		Negative	-
29	M	14.8	**49.08**	**8.76**	33.14	0.29	1.03		**Positive**	-
30	F	16.5	**11.57**	**2.07**	25.20	0.22	2.85		Negative	-
*Children with newly diagnosed isolated TSH alteration*										
31	M	12.1	9.00	0.26	11.60	0.10	**5.08**	13.67	Negative	-
32	M	14.1	0.16	0.03	11.77	0.10	**6.23**	12.51	Negative	-
33	F	15.1	0.16	0.03	17.83	0.16	**5.53**	NI	NI	-
34	F	15.8	1.07	0.19	11.96	0.10	**5.11**	12.08	NI	-
35	M	14.2	1.44	0.26	12.42	0.11	**5.30**	11.84	Negative	-
36	M	9.4	9.00	0.26	11.80	0.10	**5.11**	14.20	Negative	-
37	F	16.1	0.38	0.07	15.21	0.13	**6.45**	14.06	Negative	-
38	M	15.8	0.00	0.00	19.10	0.17	**6.97**	12.98	Negative	-
*Children with previously known thyroid abnormality*										
39	F	9.5	**92.97**	**16.60**	**489.70**	**4.26**	1.70		**Positive**	-
40	F	17.6	**2381.12**	**425.20**	**387.60**	**3.37**	**8.78**	NI	**Positive**	Levothyroxine
41	F	13.5	**208.57**	**37.24**	24.30	0.21	1.77		**Positive**	-
42	F	17.0	**1380.00**	**40.59**	55.10	0.48	0.76		**Positive**	Levothyroxine
43	M	16.3	9.70	0.29	11.70	0.10	**8.91**	20.00	NI	Levothyroxine

Non-physiological results are presented in **bold**. Ultrasound positivity means thyroiditis. Discovered ultrasound changes (*n* = 2) and newly administered medications (*n* = 1) during the follow-up period are indicated above in parentheses (negative (positive) and – (levothyroxine))

*M* Male, *F* Female, *ATPO* Anti-thyroid Peroxidase, *ATG* Antithyroglobulin, *TSH* Thyroid-stimulating Hormone, *fT4* Free Thyroxine, *NI* No Information (missing data)

^*^The patient also had TSH receptor antibody positivity

### The rate of abnormal thyroid function tests, anti-thyroid antibody positivity and ultrasound-proven thyroiditis at the first evaluation

**Fig. 2:**
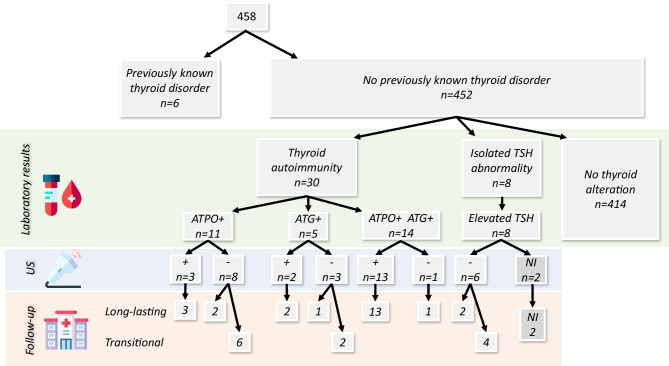
Flowchart of thyroid laboratory, ultrasound, and follow-up results. Ultrasound positivity means thyroiditis. Ultrasound outcome covers positive results diagnosed both at initial (*n* = 16) and during follow-up (*n* = 2) examinations. *ATPO *Anti-thyroid Peroxidase, *ATG* Antithyroglobulin, *TSH* Thyroid-stimulating Hormone, *US* Ultrasound, *NI* No Information (missing data)

From the descriptive analysis of thyroid laboratory markers and further comparisons, we excluded the above-mentioned six children with previously known thyroid disorders, leaving 452 included in the final analysis. Out of the 452 children, there were 25 abnormal results of ATPO (M = 254.5 U/mL, SD = 619.9 U/mL), and 19 of ATG (M = 792.5 UI/mL, SD = 1157.3 UI/mL), altogether 30 cases of newly diagnosed thyroid autoimmunity (TA) were observed (6.6%) (See Fig. 2 and Table 2). Isolated TSH abnormality without autoantibody positivity (all elevated) was seen in eight cases (M = 5.7 mU/L, SD = 0.7 mU/L). We initiated hormone replacement or anti-thyroid treatment after the first visit in three cases (See Table 2). There were 16 positive (all in children with TA), and 20 negative results out of 36 performed ultrasounds. Hence, after the first evaluation, the AIT prevalence was 3.5% among our children.

### The association between COVID-19 vaccination and thyroid autoimmunity

#### Analysis of the possible pathogenic role of vaccination

Eighty-seven (19.2%) children were vaccinated (86 of them with BNT162b2 /Pfizer-BioNTech/, 1 child with mRNA-1273 /Moderna/) by the time they arrived at our clinic. The proportion of TA was 5.7% in vaccinated, and 6.8% in non-vaccinated children. The association between vaccination and TA was non-significant (χ^2^(1,N = 452) = 0.138, *p* = 0.815) with no significant difference neither in relative ATPO (t(23) =  − 1.738, *p* = 0.098) nor in relative ATG (t(17) = 0.684, *p* = 0.561) titer results.

#### Examination of the potential preventive role of vaccination

Children who received the vaccination after their infection (*n* = 52) or had not been vaccinated at all (*n* = 365) had a 7.2% TA rate. In comparison, among those who had been vaccinated before their SARS-CoV-2 infection (*n* = 18), no TA was observed. Due to a small sample size, no statistical tests could be performed.

### The association between long COVID syndrome and thyroid autoimmunity

Thyroid autoimmunity (abnormal results of ATPO and/or ATG) and LC status (LC + and LC- group) showed a non-significant association (χ^2^(1,N = 452) = 0.342, *p* = 0.321). Positive results were 9.1% in the LC- group, and 6.1% in the LC + group.

### Thyroid autoimmunity — subgroup analyses

The proportion of girls (9.4%, *n* = 23) was higher than that of boys (3.4%, *n* = 7) in the TA group (χ^2^(1,N = 452) = 6.532, *p* = 0.013, Ф = 0.12). The association between age (< 10 yrs vs. ≥ 10 yrs) and TA was non-significant (χ^2^(1,N = 452) = 0.730, *p* = 0.504). The association between the severity of an acute infection and TA was non-significant (χ^2^(1,N = 447) = 0.262, *p* = 0.490). None of the present symptoms suggestive of thyroid dysfunction differed between TA and non-TA groups (Table 3).

**Table 3:** Presence of symptoms suggestive of hypo- or hyperthyroidsm in children with and without thyroid autoimmunity

**Symptoms**		**Non-TA, % (n)**	**TA, % (n)**		***χ***^**2**^	**p**	**Ф**
Fever	Yes	95.2 (40)	4.8 (2)	0.241		1.00	0.02
	No	93.3 (374)	6.7 (27)				
Low fever	Y	97.0 (97)	3.0 (3)	2.673		0.11	0.08
	N	92.4 (316)	7.6 (26)				
Palpitation	Y	92.5 (136)	7.5 (11)	0.305		0.68	0.03
	N	93.9 (277)	6.1(18)				
Constipation	Y	98.0 (50)	2.0 (1)	na		na	na
	N	92.9 (316)	7.1 (28)				
Diarrhoea	Y	95.0 (96)	5.0 (5)	0.535		0.65	0.04
	N	93.0 (319)	7.0 (24)				
Weight loss	Y	94.3 (99)	5.7 (6)	0.237		0.82	0.02
	N	92.9 (315)	7.1(24)				
Slowness of movement	Y	94.3 (66)	5.7 (4)	0.094		1.00	0.02
	N	93.3 (348)	6.7 (25)				
Trouble with concentrating	Y	92.0 (173)	8.0 (15)	1.004		0.34	0.05
	N	94.4 (237)	5.6 (14)				
Sleeping less	Y	91.3 (105)	8.7 (10)	1.208		0.28	0.05
	N	94.2 (311)	5.8 (19)				
Sleeping more	Y	94.4 (141)	6.6 (10)	0.003		1.00	0.00
	N	93.5 (274)	6.5 (19)				
Tremors	Y	93.9 (46)	6.1 (3)	0.016		1.00	0.01
	N	93.4 (368)	6.6 (26)				
Persistent fatigue	Y	92.7 (255)	7.3 (20)	0.360		0.70	0.03
	N	94.2 (162)	5.8 (10)				
Hair loss	Y	100.0(13)	0 (0)	na		na	na
	N	94.9(74)	5.1 (4)				

*Non-TA* children with no thyroid autoimmunity, *TA* children with thyroid autoimmunity, *Na* small sample size in cells, values cannot be calculated

### The longitudinal changes of the initial anti-thyroid positivity and abnormal thyroid function

**Fig. 3:**
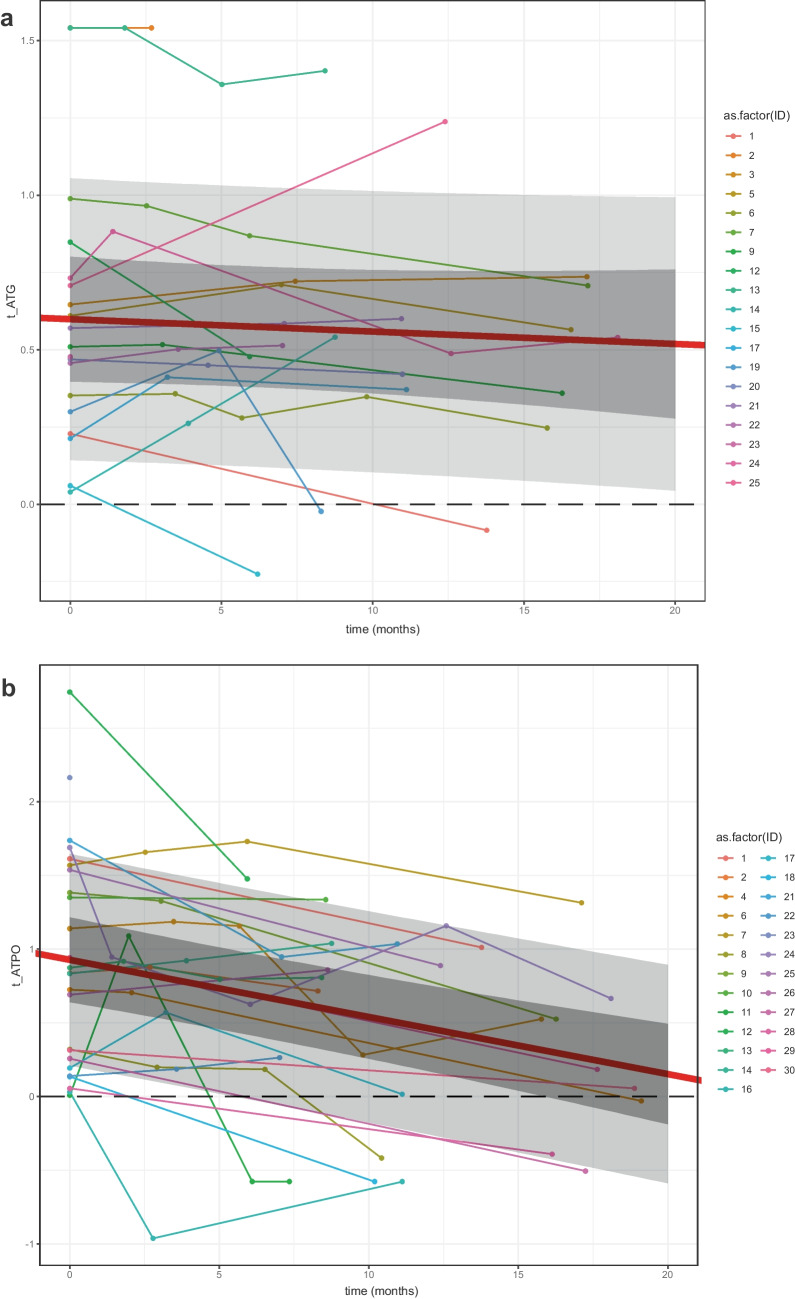
Follow-up of initial positive thyroid autoantibody results. **a** Children with initially positive antithyroglobulin. **b** Children with initially positive anti-thyroid peroxidase. Dashed black line: (a) ATG = 1, (t_ATG = log10(1)), (b) ATPO = 1, (t_ATPO = log10(1)). Wide red line: the estimated mean effect line. With intervals (inner: confidence, outer: prediction; both are 95% intervals). x-axis: time (months) y-axis: t_autoantibody = log10(1). For the estimation of the mean effect of time on autoantibody value changes, a generalized least square method was used with linear relation and autoregressive correlation structure assumptions

Of the 38 children with a newly found thyroid alteration, 36 were followed for a mean (± SD) 12.7 (± 4.3) months (See Fig. 2). Two children did not return for follow-up examinations. Results of ATPO and ATG changes over time can be seen in Fig. 3. The estimation of the mean effect of time on the autoantibody value change was significant in ATPO (β =  − 0.04[− 0.05; − 0,03], *p* < 0.001; values in base ten logarithm). The ATG value showed a non-significant change in time (β =  − 0.00[− 0.01;0.01], *p* = 0.398). No ultrasound with initially positive results became negative by time; however, two children whose original ultrasound imagings were negative, developed thyroiditis during the follow-up period (therefore, the AIT prevalence in our study group increased from 3.5 to 4.0%). One girl had to start levothyroxine treatment due to TSH elevation. Twenty-two long-lasting and eight transient disturbances were seen in children with TA. Among the children who had isolated TSH abnormality, two cases proved to have long-lasting elevated levels, four had transiently elevated levels, and two children did not return for follow-up.

## Discussion

In our multi-center prospective analysis of pediatric patients with a previous SARS-CoV-2 infection, we diagnosed 30 children with thyroid autoimmunity (6.6%) and isolated elevated TSH was discovered in eight additional cases (1.8%). The prevalence of autoimmune thyroiditis (elevated ATPO and/or ATG with positive ultrasound results) was found to be 4.0% (16 children at first visit and an additional two during the follow-up period).

As the effect of SARS-CoV-2 vaccines has been a highly discussed topic recently, we examined the possible pathogenic and/or preventive effect of vaccines on thyroid parameters among the same population (87 vaccinated children: 86 with BNT162b2 /Pfizer-BioNTech/, 1 with mRNA-1273 /Moderna/; 365 non-vaccinated) focusing on two aspects. As all of these children had been through COVID-19, the effect of vaccination could be evaluated in a homogenous group, with vaccinated and unvaccinated children being a control group for each other. First, to determine whether vaccines could have a pathogenic role (cross-reactivity between thyroid cell antigens and the viral spike protein or autoimmune/inflammatory syndrome induced by adjuvants [ASIA]), as proposed by several studies [8, 17–20]; we compared the prevalence of TA and ATPO and ATG titers in vaccinated and unvaccinated children. The association between vaccination and the above-mentioned parameters was non-significant, which means that children who had been vaccinated before they arrived at our clinic did not have a greater chance of developing TA. Our second goal was to examine the potential preventive effect of vaccines. Although we could not perform statistical tests due to the small sample size, it is worth mentioning that among the 18 children vaccinated before their acute SARS-CoV-2 infection, no TA was observed in our study. In conclusion, we did not find an increased risk for TA in vaccinated children compared to unvaccinated ones. A population-based study also found no increase in vaccine-related hyperthyroidism or hypothyroidism in adults [21].

Additionally, in line with our hypothesis of thyroid (or any) alterations being more frequent in children with suspected LCS, we analyzed children with persisting symptoms after COVID-19 (LC +) and those who were complaint-free at our examination (LC-) separately. No significant difference was found between the two groups, therefore, according to our analysis, thyroid autoimmunity has no role in LC among children.

To investigate different factors that might influence the presence of TA, we performed several subgroup analyses. Thyroid autoimmunity was found to be more frequent among girls, in line with prior voluminous data [22–24]. However, in contrast with some previous studies [22, 24], age showed no effect on the rate of autoimmune thyroid alterations in our study. As we pointed out earlier, thyroid abnormalities can frequently occur among hospitalized patients with COVID-19 [25, 26]; however, according to our dataset, the severity of acute COVID-19 did not influence the prevalence of subsequent TA. It should be noted that children with LCS might have complaints which can be easily confused with symptoms of either hypo- or hyperthyroidism. The large majority of our children with TA had no alterations in their fT4 levels and — in line with these normal laboratory results — their symptoms were also similar when compared to those without TA. In conclusion, screening children with symptoms suggestive of thyroid dysfunction only is insufficient to recognise all TA and AIT cases, meanwhile other health conditions (e.g., LCS) can mimic these diseases, therefore a careful evaluation is needed.

The prevalence of autoimmune thyroid disorders varies between regions and are influenced by different genetic and environmental factors [27, 28]. As we do not have a control group, we aim to interpret our results of 6.6% of TA and 4.0% of AIT (7.4% and 4.8% when children with previously known thyroid disorders were also included) in light of the healthy European pediatric population. Kondrashova et al. found 2.4% positivity of ATPO and/or ATG in Finnish and Russian Karelian schoolchildren (with a significant difference between Finland [4.3%] and Russian Karelia [0.6%]) [24]. Also, TA was found to be 3.7% in Spanish children without a known endocrinological disorder [22]. In a Greek study, the prevalence of AIT was 2.5% [29].

A few pediatric case reports have described children whose autoimmune thyroid disorder was recognised after their SARS-CoV-2 infection [30–33]. The proposed pathophysiology of COVID-19-induced thyroid disturbances according to voluminous recent publications is that SARS-CoV-2 might be able to cause thyroid dysfunction through direct cell damage (by entering through angiotensin-converting [ACE-2] receptors) or indirect mechanisms (autoimmunity triggering or accelerating effect) [34–36].

When assessing permanence, the majority (73.3%) of children with TA had long-lasting alterations, with some cases even progressing into AIT. All children with both ATPO and ATG positivity and/or positive ultrasound results sustained long-lasting alterations.

As we stated before, our main limitation is the lack of pre-pandemic data on thyroid autoantibodies of these children or the general pediatric population in Hungary. Since by now — according to our clinical experience — most children have already contracted SARS-CoV-2, a currently initiated study would hardly give us an answer. It should also be noted that it is probable that a considerable number of these children already had these alterations before their COVID-19, as lacking abnormal fT4 values, they were asymptomatic, and therefore did not seek medical help earlier. Another limitation was that the laboratory test method used for the measurement of ATPO was changed during our study period, therefore we calculated and illustrated the relative ATPO values. Although two different autoantibody tests never give perfectly matching results, we believe that we found an acceptable solution with this calculation and representation.

What makes our study unique is the systematic testing of more than 450 children for TA and other thyroid dysfunctions after COVID-19 with detailed analyses of possible risk factors, such as vaccination, and also the temporality of the found alterations.

In conclusion, as in our multi-center, prospective study, we did not find any evidence of increased thyroid autoimmunity rate after COVID-19 vaccination, the BNT162b2 /Pfizer-BioNTech/ vaccine has proved to be safe regarding TA in children. According to our analysis, thyroid autoimmunity has no role in LC among children. Among pediatric patients with previous SARS-CoV-2 infection, we discovered a slightly higher rate of thyroid autoimmunity and autoimmune thyroiditis than in the average pediatric population. The majority of these alterations remained positive or even progressed into thyroiditis; therefore, until controlled studies state otherwise, thyroid screening and in case of positivity, regular, long-term follow-up after COVID-19 should be considered in order to early recognise hormonal complications and malignancies. Further evaluation is needed using both experimental and clinical studies to comprehensively understand the patophysiology of the potential COVID-19-induced thyroid disturbances.

## Data Availability

The datasets generated and analyzed during the current study are available from the corresponding author on reasonable request.

## Abbreviations

ACE-2: Angiotensin-converting enzyme 2
AIT: Autoimmune thyroiditis
ASIA: Autoimmune/inflammatory syndrome induced by adjuvants
ATG: Antithyroglobulin
ATPO: Anti-thyroid peroxidase
COVID-19: Coronavirus disease 2019
fT4: Free thyroxine
LC: Long COVID
LCS: Long COVID syndrome
PCR: Polymerase chain reaction
RAT: Rapid antigen test
SARS-CoV-2: Severe acute respiratory syndrome coronavirus 2
SAT: Subacute thyroiditis
TA: Thyroid autoimmunity
TRAb: TSH receptor antibodies
TSH: Thyroid-stimulating hormone
WHO: World Health Organization
